# Lactate-induced MRP1 expression contributes to metabolism-based etoposide resistance in non-small cell lung cancer cells

**DOI:** 10.1186/s12964-020-00653-3

**Published:** 2020-10-23

**Authors:** Qi Dong, Chenkang Zhou, Haodong Ren, Zhijian Zhang, Feng Cheng, Zhenkai Xiong, Chuantao Chen, Jianke Yang, Jiguang Gao, Yao Zhang, Lei Xu, Jian Fang, Yuxiang Cao, Huijun Wei, Zhihao Wu

**Affiliations:** 1 School of Clinical Medicine, Wannan Medical College, Wuhu, 241001 China; 2 Research laboratory of Tumor Microenvironment, Wannan Medical College, Wuhu, 241001 China; 3 Anhui Province Key laboratory of Active Biological Macro-molecules Research, Wannan Medical College, Wuhu, 241001 China; 4 Anhui provincial Engineering Research Center for Polysaccharide Drugs, Wannan Medical College, Wuhu, 241001 China; 5 School of laboratory Medicine, Wannan Medical College, Wuhu, 241001 China; 6 School of pharmacy, Wannan Medical College, Wuhu, 241001 China; 7 School of Preclinical Medicine, Wannan Medical College, Wuhu, 241001 China; 8 School of Medical Imageology, Wannan Medical College, Wuhu, 241001 China

**Keywords:** Metabolic reprogramming, Chemoresistance, Lactic acid, MRP1, Etoposide

## Abstract

**Background:**

Metabolic reprogramming contributes significantly to tumor development and is tightly linked to drug resistance. The chemotherapeutic agent etoposide (VP-16) has been used clinically in the treatment of lung cancer but possess different sensitivity and efficacy towards SCLC and NSCLC. Here, we assessed the impact of etoposide on glycolytic metabolism in SCLC and NSCLC cell lines and investigated the role of metabolic rewiring in mediating etoposide resistance.

**Methods:**

glycolytic differences of drug-treated cancer cells were determined by extracellular acidification rate (ECAR), glucose consumption, lactate production and western blot. DNA damage was evaluated by the comet assay and western blot. Chemoresistant cancer cells were analyzed by viability, apoptosis and western blot. Chromatin immunoprecipitation (ChIP) was used for analysis of DNA-protein interaction.

**Results:**

Here we showed that exposure to chemotherapeutic drug etoposide induces an exacerbation of ROS production which activates HIF-1α-mediated the metabolic reprogramming toward increased glycolysis and lactate production in non-small cell lung cancer (NSCLC). We identified lactic acidosis as the key that confers multidrug resistance through upregulation of multidrug resistance-associated protein 1 (MRP1, encoded by *ABCC1*), a member of ATP-binding cassette (ABC) transporter family. Mechanistically, lactic acid coordinates TGF-β1/Snail and TAZ/AP-1 pathway to induce formation of Snail/TAZ/AP-1 complex at the MRP1/ABCC1 promoter. Induction of MRP1 expression inhibits genotoxic and apoptotic effects of chemotherapeutic drugs by increasing drug efflux. Furthermore, titration of lactic acid with NaHCO_3_ was sufficient to overcome resistance.

**Conclusions:**

The chemotherapeutic drug etoposide induces the shift toward aerobic glycolysis in the NSCLC rather than SCLC cell lines. The increased lactic acid in extracellular environment plays important role in etoposide resistance through upregulation of MRP expression. These data provide first evidence for the increased lactate production, upon drug treatment, contributes to adaptive resistance in NSCLC and reveal potential vulnerabilities of lactate metabolism and/or pathway suitable for therapeutic targeting.

Video Abstract

**Graphical abstract:**

**Figure:**
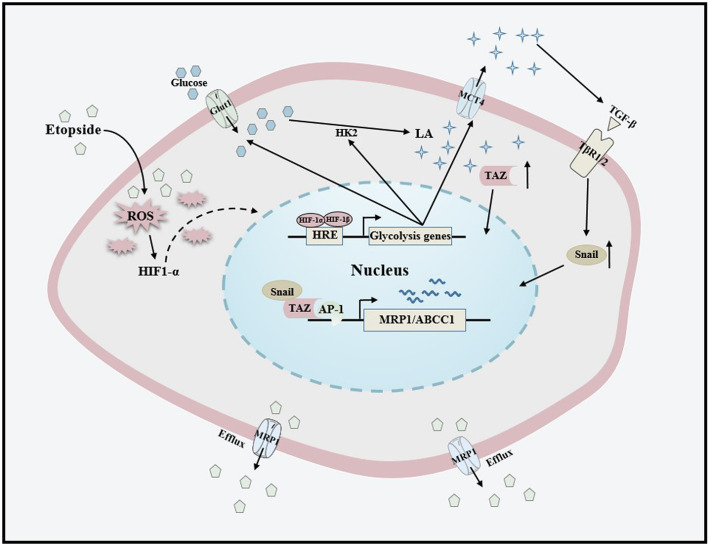
The chemotherapeutic drug etoposide induces metabolic reprogramming towards glycolysis in the NSCLC cells. The secreted lactic acid coordinates TGF-β1/Snail and TAZ/AP-1 pathway to activate the expression of MRP1/ABCC1 protein, thus contributing to chemoresistance in NSCLC.

## Background

Despite the advent of targeted therapy and recent breakthrough of immunotherapeutics, lung cancer remains the most prevalent fatal malignancy worldwide. Although the recent therapeutic advances have provided significant survival benefits of some selected patients with non-small cell lung cancer (NSCLC), approximately 60% NSCLC do not have targetable driver mutations, and only 20% NSCLC patients might respond to anti-PD-1 or PD-L1 treatment [1–3]. Understandably, systemic chemotherapy is still the mainstay of NSCLC treatment. However, the relatively rapid emergence of resistance substantially limits our ability to treat lung cancer. Therefore, better understanding of molecular determinants of chemotherapy resistance is a critical challenge to improve the outcome of patients with such treatment strategies. While substantial work has sought to define genetic or epigenetic alterations manifest in acquired resistance, little attention has been given to non-cell-autonomous mechanisms of resistance. Recent work has established equally important roles of tumor microenvironment in tumor progression, and might also in treatment responses. Indeed, several studies have shown that hypoxia and factors derived from tumor microenvironment can play a critical role in resistance onset [4–7].

One of characteristic hallmarks of cancer is the reprogramming of cellular metabolism that contributes tumor development and chemotherapy failure [8–10]. In contrast with their normal counterparts, cancer cells reprogram the metabolism to primarily rely on aerobic glycolysis even in normoxic conditions. This phenomenon, known as the Warburg effect, allows cancer cells to fine tune their energetic demands and precursor materials to sustain high proliferative rates. A further advantage of the high glycolytic rate is a characteristic increase in lactate production, which, in turn, accumulate in its extracellular environment. The high levels of lactate accumulation in tumor microenvironment has been associated with certain key features of tumor progression including senescence suppression, invasion and immune evasion [11–13], however, the role of lactate in chemoresistance has been relatively unexplored.

It is increasingly clear that lactate is emerging as a key player in carcinogenesis. In tumor microenvironment lactate levels could elevate up to a 40-fold and are highly correlated with distant metastasis and poor prognosis [14, 15]. While much of the literature considers lactate as end-product as a consequence of tumor metabolic reprogramming, accumulating evidence, however, indicates that lactate also functions as a signaling molecule that coordinates many steps in carcinogenesis, including angiogenesis, cell migration, metastasis and self-sufficiency of cancer stem cells [16, 17]. It has been recently reported that lactate induced by drug treatment sustains the acquisition of resistance to certain targeted therapies [6]. In this regard, drug treatment induces a fast adaptation of cellular metabolism, and then secondarily leads to drug resistance. However, it is entirely unknown how tumor cells undergoing chemotherapy can reprogram their metabolism to promote chemoresistance. Thus, we set out to determine whether metabolic reprogramming upon drug treatment could instruct drug resistance onset. Etoposide, a frontline chemotherapeutic drug for lung, testicular and ovarian cancers, is an inhibitor of topoisomerase II enzyme that resulted in double-stranded DNA breaks (DSB). There is lack of understanding as to why NSCLC is more resistant to etoposide than small cell lung cancer (SCLC) [18].

Previously, we found that lactic acid activates Snail expression, a major inducer of EMT, by directly remodeling extracellular matrix and releasing activated TGF-β1 [11]. We also demonstrated that lactic acid increased transcriptional co-factor TAZ expression and activity in lung cancer cells [19]. In the present study, we report that drug treatment induced lactate secretion by shifting cellular metabolism toward glycolysis in NSCLC. The increasing aerobic lactate production confers a potent chemoresistance through the upregulation of ATP-binding cassette (ABC) transporter MRP1 expression, which contributes to drug resistance by pumping the drugs out of cells. Furthermore, we demonstrated that lactic acid-induced upregulation of Snail and TAZ form complexes with AP-1 transcriptional factor to activate MRP1 expression. Finally, we showed that neutralization of lactic acid with NaHCO_3_ rescues sensitivity of cancers to drug exposure, indicating the clinical relevance of our findings. Cumulatively, our data suggest that the increased lactate production, upon drug treatment, contributes to adaptive resistance in NSCLC via feedback mechanisms.

## Materials and methods

### Cell culture, antibodies, reagents and plasmids

A549 (human lung adenocarcinoma), H1299 (human lung adenocarcinoma) and H446 (human small cell lung cancer) cells were cultured with DMEM (Hyclone, Logan, UT, USA) containing 10% fetal bovine serum (FBS, GibcoBRL, Grand Island, NY, USA) at 37 °C in a humidified atmosphere of 5% CO2. Anti-ATM (873), Anti- Phospho-ATM (Ser-1981) (13050), Anti-PARP (9542), Anti-p53(2527), Anti-Snail (3895), Anti-Puma (4976), Anti-YAP/TAZ (8418), Anti-LDHA (3582), Anti-Phospho-Smad2 (3101), Anti-Phospho-Smad3 (9520), were all obtained from Cell Signaling Technology (Danvers, MA, USA). Anti-MRP1 (K003560P), Anti-HK2 (K001797P) were obtained from Solarbio (Beijing, China). anti-MCT1 (sc-365,501) were obtained from Santa Cruz Biotechnology (Santa Cruz, CA, USA). Anti- SLC16A4 (MCT4) (A3016), Anti-GLUT1 (A6982) were obtained from Abclonal (Wuhan, China). Anti-β-Actin (A1978) was obtained from Sigma-Aldrich (St. Louis, USA), Anti-gamma H2A.X (ab11174), anti-CTGF (ab6992) and Anti-GPR81 (ab124010) were purchased from Abcam (Cambridge, UK). Twenty milligram/milliliter Etoposide solution (H20143143) was obtained from QILU PHARMACEUTICAL Co.,Ltd. (Hainan, China). Five milligram/milliliter Cisplatin solution (H20040813) was purchased from HANSOH PHARMA (Jiangsu, China). Six milligram/milliliter Paclitaxel solution was obtained from SL PHARM (Beijing, China). One milligram/milliliter Doxorubicin Hydrochloride was obtained from Shenzhen Main Luck Pharmaceuticals Inc. (Shenzhen, China). HA-TAZ (32839), Flag-Snail (16218), p53 (16343) plasmids were purchased from Addgene (Cambridge, MA, USA). pAP-1-Luc (D2108) plasmid was purchased from Beyotime (Shanghai, China), HRE-Luc (9PIE400) plasmid was purchased from Promega (Madison, WI, USA).

### Cloning and DNA construction

To construct different length of MRP1/ABCC1 promoters, fragments were amplified from genome DNA of BEAS-2B (B2B, normal lung epithelial) cells by PCR and were then cloned into pGL3-Basic Vector (Promega, Madison, WI, USA) at the Kpn I and Hind III sites. Point mutations in the MRP1/ABCC1 promoter were generated by site-specific mutagenesis using the overlap PCR extension method and the longest MRP1/ABCC1 promoter was used as the template. The primers are listed in Table 1.

**Table 1:** Primers used for PCR amplifications

Gene	Genebank accession number	Primer (5′-3′)
ABCC1	NM_004996	Fwd:AACAATCCTATCTACCTTCCTCCT Rev.:CCACACACCCTGCGACCACTTTTCAA
ABCC1	NM_004996	Fwd:GGCTGCTCACGGGTTTGAATCT Rev.:CCACACACCCTGCGACCACTTTTCAA
ABCC1	NM_004996	Fwd:AACAATCCTATCTAACGGACTCCT Rev.:CCACACACCCTGCGACCACTTTTCAA
ABCC1	NM_004996	Fwd:TTAAGGCGCGTATGCGAAAGGCAGGTAGGGGGCT Rev.:AGCCCCCTACCTGCCTTTCGCATACCGGGCGCCTTAA
ABCC1	NM_004996	Fwd:GGTACC CCTTCTGT GTGACTCAGC Rev.:CCACACACCCTGCGACCACTTTTCAA
ABCC1	NM_004996	Fwd:GGTACC CCTTCTGT TGCGTATTGC Rev.: CCACACACCCTGCGACCACTTTTCAA
ABCC1	NM_004996	Fwd:AACAATCCTATCTACCTTCCTCCT Rev.:CCACACACCCTGCGACCACTTTTCAA

### Cell viability assay

Cell viability was determined using a 3-(4,5-dimethylthiazole-2-yl)-2.5-diphenyl tetrazolium bromide (MTT) (Beyotime Institute of Biotechnology, Shanghai, China) assay. The range of etoposide concentrations was selected to obtain concentration for 50% of maximal effect (EC_50_) for treated cells. After treatment, cells were washed with PBS and incubated with fresh medium with MTT solution (0.5 mg/ml) for 4 h at 37 °C, the resulting formazan crystals were dissolved in DMSO and absorbance at wavelength of 490 nm was taken on a plate reader using BioTek citation 5 (BioTek, Winooski, VT). The measured absorbance value (OD) from the cells incubated without drugs were used for 100% survival and the inhibition rate of samples were calculated, via the formula:

The Inhibition rate = ((OD_control_-OD_experiment_)/(OD_control_)) × 100%. We then used the inhibition rate at each point to calculate EC50 values using SPSS 26.0 software package (www.ibm.com). For the viability of transfected cells, cells were first transfected with corresponding clones. After transfection, cells were treated with etoposide followed by MTT assay at different concentration points. Each point performed in triplicate.

### Lactate dehydrogenase cytotoxicity assay

Lactate dehydrogenase assay was performed according to the manufacturer’s instructions (11,644,793,001; Cytotoxicity Detection Kit, Roche Mannheim, Sandhofer Strae, Germany). The absorbance of the samples was measured at wavelength of 492 nm using a microplate reader.

### Lactate determination

Cells (2 × 10^5^) were treated with Etoposide for 5 h. Lactate in the Culture medium was measured using the Lactate Assay Kit (BioVision, Milpitas, CA, USA) according to the manufacturer’s instructions. The concentration of lactate was determined using Lactate Standard Curve.

### Plasmid and short interfering RNA (siRNA) transfection

Cells seeded in plates were grown to 70–90% confluence before plasmids transfection and transfection of plasmids was done with PolyJet DNA Transfection Reagent (Signa-Gen Laboratories, Gaithersburg, MD, USA) according to the manufacturer’s instructions. The transfection with siRNA was performed by using GenMute siRNA Transfection Reagent (Signa-Gen Laboratories) when cells seeded in plates were grown to 30–50% confluence. All the siRNAs were purchased from RiboBio Company (Guangzhou, China). After transfection for 48 h, cells were deprived of serum and growth factors for 12 h and then treated with lactic acid (Roche, San Francisco, CA, USA) for 3 h and harvested. The sequences of the siRNAs are listed in Table 2.

**Table 2:** Sequences of siRNA

Gene	Genebank accession number	Target sequence (5′-3′)
siLDHA	NM_001165414.1	GCCAUCAGUAUCUUAAUGATT
siGPR81	NM_032554.3	CTGCTAGACTCTATTTCCT
siABCC1	NM_004996	GAUGACACCUCUCAACAAAdTdT
siTEAD1	NM_021961.6	GCCCUGUUUCUAAUUGUGGTT
siHIF-1α	NM_001530	CCAGCAGACUCAAAUACAATT
siSnail	NM _005985.3	CAAATACTGCAACAAGGAA
sic-Jun	NM_002228	AAGAACGTGACAGATGAGCAG

### Chromatin immunoprecipitation (ChIP) assay

The Cells were transfected with Snail cDNA. ChIP were performed using the Simple-ChIP Enzymatic Chromatin IP kit (Cell signaling technology, No. 9002) according to the manufacturer’s instructions with minor modifications at 72 h post-transfection. The cells were fixed in DMEM medium containing 1% formaldehyde for 15 min at room temperature, and the reaction was stopped by glycine quenching (125 mM final concentration). Nuclei were collected and digested with micrococcal nuclease (provided by the Simple-ChIP kit) followed by 2 min of sonication (3 cycles of 10 s of sonication and 30 s without sonication) using a vibra cell VCX 130 (Sonics & Materials, Inc., NEWTOWN, CT, USA). Pull downs were performed on DNA fragments (ranging from 150 to 900 bp) using anti-FLAG (M2) antibody (SIGMA, No. F1804). The immunoprecipitated DNA and input DNA were extracted by reversing the crosslinks. Standard PCR and qRT-PCR were performed with purified DNA as templates. The primers used were: MRP1/ABBC1 forward: 5′- CCTTCTGT GTGACTCAGC-3′; reverse:5′- ACTCCAAAGCTGAGTCACACAGAAGG-3′. The standard PCR products were run on a 1% agarose gel and scanned under UV using FluorChem FC3 (ProteinSimple, San Jose, CA, USA), and qRT-PCR results were analyzed according to the protocols.

### Dual luciferase reporter assays

Cells were co-transfected with experimental reporter constructs. After 48 h transfection, cells were lysed and activities of firefly luciferase and Renilla luciferase were analyzed following the manufacturer’s instruction. Each experiment was repeated in triplicate using a multimode microplate reader (TriStar LB941; Berthold Technologies, Bad Wildbad, Germany). The results were expressed as mean of triplicates ± SD.

### Western blot

Cells were scraped and homogenized with Sample Buffer, Laemmli (2 x Concentrate, S3401; SIGMA). The homogenate was separated by polyacrylamide gel electrophoresis and then transferred to nitrocellulose (NC) membrane (GE Healthcare, Piscataway, NJ, USA) and detected with the antibodies. The antibody dilution ratios were referred to the respective companys’ instruction manuals. Except Anti-β-actin was diluted with 5% skim milk at a ratio of “1:5000″, the remaining antibodies were all diluted with 5% skim milk at a ratio of “1:1000″. The signals were scanned by FluorChem FC3 (ProteinSimple, San Jose, CA, USA).

### Hoechst 33342 efflux assays

The cells were first treated with or without lactate for 6 h, then the cells were rapidly washed with PBS, and the medium was replaced with fresh medium with Hoechst dye (10 μg/ml) in the presence and absence of the inhibitor of verapamil (10 μM), the cells were further incubated for 10 min at 37 °C in the incubator. Subsequently the Hoechst medium was removed and the fluorescence intensity was quantitatively analyzed using BioTek citation 5 (BioTek, Winooski, VT).

### Comet assays

Cells (2 × 10^5^) were pretreated with lactate for 3 h, then treated with lactic acid and etoposide for 36 h. The comet assay was performed using the reagents from the OxiSelect Comet assay kit (Cell Biolabs, San Diego, CA, USA) according to the manufacturer’s instructions. Comets were viewed using BioTek citation 5 (BioTek, Winooski, VT, USA) in the fluorescein GFP channel using a 20x and 4x objective. The DNA% in Comet Tail were measured using Software Gen5 (BioTek, Winooski, VT, USA).

### Flow cytometry

Cells (2 × 10^5^) were first stimulated with lactic acid for 3 h, then treated with etoposide in the presence and absence of lactic acid for 36 h. Samples preparation was performed using the reagents from the BD Pharmingen FITC AnnexinV Appotosis Detection Kit (556,547, Becton Dickinson, USA) according to the manufacturer’s instructions. All flow cytometric analyses were performed using a BD FACSVerse Flow Cytometer (Becton Dickinson, USA).

### Co-immunoprecipitation

For co-immunoprecipitation experiments, the cells were seeded in 100 mm diameter of petri dishes at 80–90% of confluence the day before transfection. The following day, plasmids of pcDNA3.1, Snail-flag, HA-TAZ were transfected 5 μg using Polyjet according to the manufacturer’s guidelines. After 48 h transfection, the cells were harvested and lysed in 1 ml of protein lysis buffer (20 mM Tris-HCl (pH 7.4), 150 mM NaCl, 1 mM EDTA,1% Triton-X100) supplemented with protease inhibitor (Roche, Switzerland). Protein lysates were incubated with 2 μg of anti-HA/flag antibody and 30 μl of protein A/G plus agarose (Santa Cruz Biotechnology) in 1.5 ml eppendorf tubes and rotated at 4 °C for overnight incubation. The beads were then washed three times using PBS. After wash, 30 μl of the 2xLaemmli sample buffer (Bio-Rad, USA) was added into the beads and then boiled for 5 min. The supernatants from each sample were then subjected to western-blotting for detection of either Flag tagged protein or HA-tagged protein. Anti-HA antibody were purchased from Cell Signaling Technology (3724S). Anti-Flag was purchased from Sigma (F1804). A standard western-blot protocol was used.

### Quantitative real-time RT–PCR analysis

Cells were treated as indicated and total mRNA was isolated using TRIzol according to the manufacturer’s protocols. The obtained RNA was re-transcribed using PrimeScript First Strand cDNA Synthesis Kit (TaKaRaBio, DaLian, China). The cDNA was mixed with ABISYBR Green Master Mix (Applied Biosystems, Carlsbad, CA, USA), and the mixture was subjected to amplification using an ABI 7500 Real-time PCR System (Applied Biosystems). The primers are listed in Table 3.

**Table 3:** Primers used for RT-PCR

Gene	Genebank accession number	Primer (5′-3′)
*ABCA2*	NM_212533	Forward: ATGTCAGCCTGCAAGAGGTG Reverse: AGATCTGGGAGAAGAGTGCC
*ABCC1*	NM_004996	Forward: AGCCGGTGAAGGTTGTGTAC Reverse: TGACGAAGCAGATGTGGAAG
*ABCC3*	NM_003786	Forward: CCTTTGCCAACTTTCTCTGC Reverse: AGGGCACTCAGCTGTCTCAT
*ABCC5*	NM_005688	Forward: ACCCGTTGTTGCCATCTTAG Reverse: GCTTTGACCCAGGCATACAT
*ABCG2*	NM_001348985	Forward: GCAGATGCCTTCTTCGTTATG Reverse: CTGTCACAGTGGCCGTCACT

### Metabolic assays

The cells were seeded at 40,000 cells per well in a 24-well cell culture XF microplate (Seahorse Biosciences) and allowed to adhere for 24 h. Then the cells were treated with etoposide for 5 h. Cells were rinsed with assay medium (unbuffered DMEM supplemented with 10 mM glucose and 20 mM sodium pyruvate, pH 7.4) before incubation with assay medium for 1 h at 37 °C in a non-CO2 incubator. The oligomycin and FCCP were sequentially injected into each well according to the manufacturer’s standard protocol. The changes in pH were determined by the sensor probe on an XF24 extracellular flux analyzer (Seahorse, Agilent Technologies). Results were normalized to the protein content of each well. The experiment was repeated five times (*n* = 5) with adequate technical replicates.

### Fluorescence microscope analysis of reactive oxygen species (ROS)

Cells (2 × 10^5^) were treated with etoposide for 5 h. Then the reactive oxygen species (ROS) was measured using Reactive Oxygen Species Assay Kit (Beyotime, Shanghai, China) according to the manufacturer’s instructions.

### Transcriptome data analyses in human NSCLC tissues

The gene correlations were analyzed using the Cancer Genome Atlas (TCGA) data (RNA-Seq-HTSeq-FPKM-UQ) in Lung adenocarcinoma (*n* = 533), Lung squamous (*n* = 534) (http://tcga-data.nci.nih.gov) and GEO database (https://www.ncbi.nlm.nih.gov/ GSE101929). Subsequently, the Spearman’s rank correlation coefficient (rho) between SNAI1 gene expression and MRP1 was calculated. All statistical analyses and data generation were carried out using R version 3.4.3 (http://www.r-project.org).

### Statistical analysis

Statistical analyses were performed with analysis of variance (ANOVA) using SPSS 13.0 Statistical Software (SPSS Inc., Chicago, IL, USA) and are presented as mean ± SD from triplicated independent experiments. A significant difference was considered when the *P*-value from a two-tailed test was < 0.05.

## Results

### Induction of lactate metabolism inhibits genotoxic and apoptotic effects of etoposide in NSCLC

**Fig. 1:**
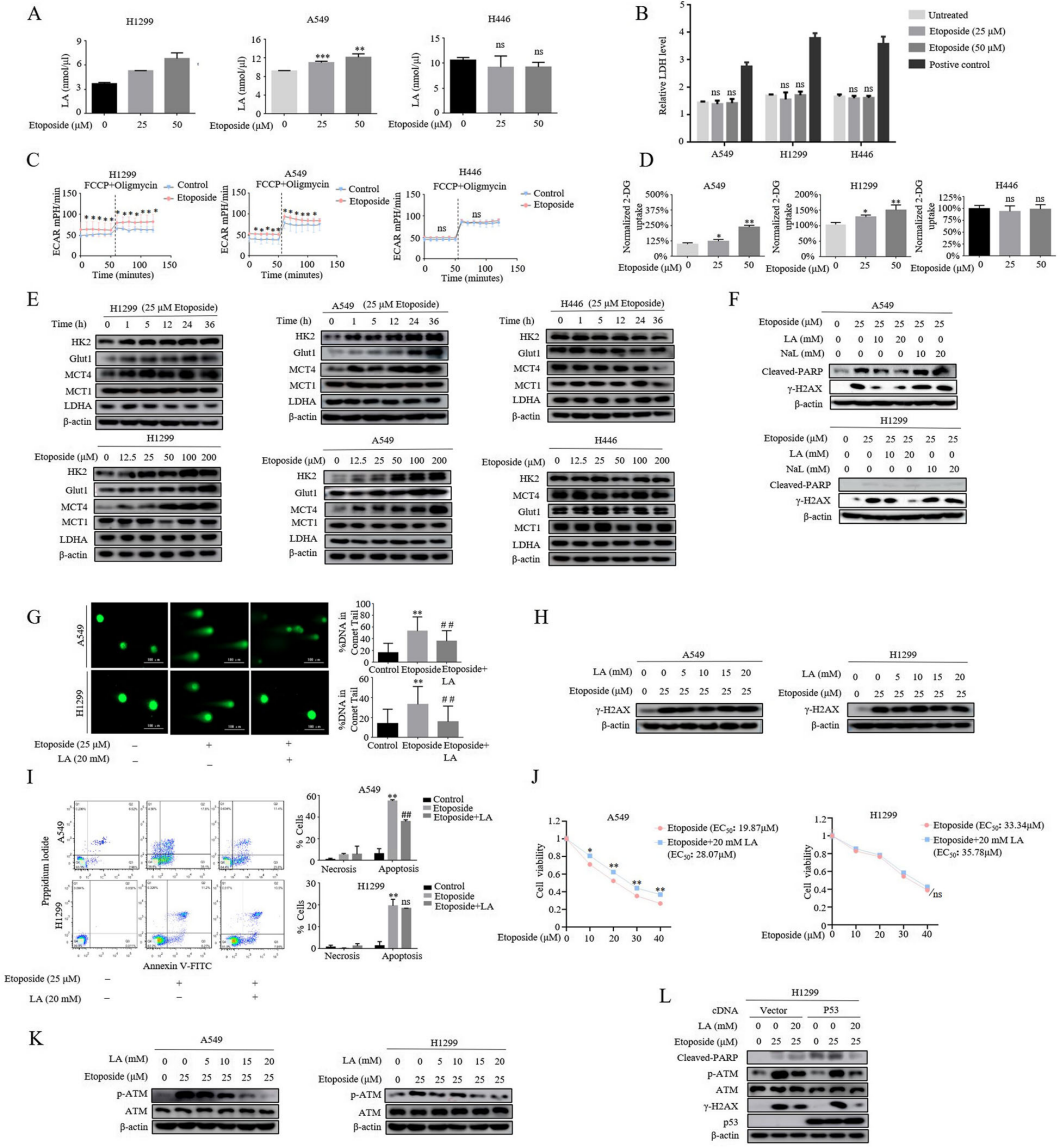
Induction of lactate metabolism inhibits genotoxic and apoptotic effects of etoposide in NSCLC. **a** Treatment of cells with etoposide for 5 h resulted in an increase in lactate secretion (*p<0.05, **P<0.01, ***p<0.001 for difference from untreated control by ANOVA for multiple comparison, ns means no statistical difference). **b** The cells were treated with different concentrations of etoposide or 1% Triton X-100 as positive control for 5 h. Culture medium was collected for determination of released lactate dehydrogenase (LDH) reflecting the membrane rapture. No significant difference of LDH release was detected after etoposide treatment (difference from untreated control by ANOVA for multiple comparison, ns means no statistical difference). **c** The extracellular acidification rate (ECAR) was measured in cells exposed to 25 μM etoposide for 5 h. The concentrations of oligomycin, FCCP were 10 μM and the bar is representative of mean ± S.D. of three independent experiments (*p<0.05, student t-test). **d** Measurement of 2-DG uptake in cells after exposed etoposide for 5 h. (*p<0.05, **P<0.01, for difference from untreated control by ANOVA for multiple comparison, ns means no statistical difference). **e** Western blot analysis was performed to examine glycolysis enzymes, HK2, MCT4, LDHA, MCT1 and Glut1, in different histological lung cancer cell lines A549, H1299 and H446 treated for indicated time (upper panel, 25 μM) or for various concentrations (lower panel) of etoposide. **f** The cells were first stimulated either with LA (lactic acid) or NaL (sodium lactate) for 3 h followed by further treatment with 25 μM etoposide for additional 36 h and then lysed for western blot analysis using antibodies against Cleaved-PARP and γH2AX. It is lactic acid, but not its sodium salt, suppressed the levels of γH2AX in both A549 and H1299 cell lines. **g** Comet assay reveal that 20 mM LA (lactic acid) inhibited DNA damage induced by etoposide. The quantification was present in right panel. The bars represent the mean ± S.D. of triplicates (**p<0.01 for difference from control cells, ^##^*p*<0.01 for difference from etoposide-treated cells by ANOVA with Dunnett’s correction for multiple comparisons). **h** A549 and H1299 were incubated first with etoposide for 5 h and then subjected to LA (lactic acid) exposure in the presence of etoposide for additional 36 h. The results revealed that etoposide induced γ-H2AX was not inhibited by lagging treatment with LA. **i** A549 and H1299 cells were pre-treated with LA (20 mM) for 3 h before etoposide treatment for 36 h. Flow cytometric analysis of cell death showed that lactate significantly reduced etoposide-induced apoptosis in A549 but not in H1299 cells. The quantification was present in right panel. The bars represent the mean ± S.D. of triplicates (**p<0.01 for difference from control cells, ^##^*p*<0.01 for difference from etoposide-treated cells by ANOVA with Dunnett’s correction for multiple comparisons, ns means no statistical difference). **j** Cell viability assay showed that LA significantly increased A549 rather than H1299 cell viability. In A549 cells, the EC_50_ (concentration for 50% of maximal effect) of etoposide was 19.87 μM, the cell survival was increased in the presence of LA with EC_50_ of 28.07 μM. However, the LA stimulation had not effect of EC_50_ values in H1299 cells. (*p<0.05, for difference from untreated control by ANOVA with Dunnett’s correction for multiple comparisons). **k** Cells were treated with different concentrations of LA for 3 h in the absence and presence of 25 μM etoposide. ATM and p-ATM expression were examined by western blot. **l** 24 h after transfection with wild-type p53 plasmids, cells were treated with etoposide in the absence and presence of 20 mM LA. The expression levels of Cleaved-PARP, γ-H2AX, ATM, p-ATM were analyzed by western blot

We noted that elevated secretion of lactate induced by etoposide (VP-16), widely used in the chemotheraputic treatment of lung cancer but possess different chemo-sensitivity and efficacy towards SCLC and NSCLC, was observed in NSCLC rather than SCLC cells (Fig. 1a). The high levels of lactate in the culture medium was not due to leakage caused by membrane rupture as determined by analyzing the release of lactate dehydrogenase. No differences in LDH release was detected following etoposide exposure at this analyzed time point (Fig. 1b). It has been classically described that NSCLC cell lines were more resistant to topoisomerase II inhibitors than SCLC cell lines. Given that cancer metabolism is intimately linked to drug resistance, we considered the possibility that drug treatment might induce fast adaptation of cellular metabolism in NSCLC, which in turn contribute the acquired resistance to chemotherapy. To test this hypothesis, we first monitored bioenergetic profiling by performing a real-time extracellular flux analysis in lung cancer cells treated with etoposide at doses commonly used in cancer cells. Notably, the extracellular acidification rate (ECAR), a measure of glycolysis, was significantly increased in etoposide-treated A549 and H1299 cells (Fig. 1c). Importantly, we did not observe notable change in glycolysis (EACR) in SCLC cell line H446 cells (Fig. 1c). Consistently, glucose consumption was accelerated only in etoposide-treated NSCLC (Fig. 1d), indicating drug-induced radical shift toward aerobic glycolysis (the Warburg effect) in the NSCLC cells.

The findings that reprogramming of glucose metabolism by etoposide prompted us to investigate whether etoposide regulates the expression of genes related to glycolysis such as Hexokinase 2 (HK2), lactate dehydrogenase A (LDHA), glucose transport 1 (Glut1), Monocarboxylate transport 4 and 1 (MCT4 and MCT1), and we found that HK2, Glut1 and MCT4 were strongly induced by etoposide in A549 and H1299 cells in a dose-dependent manner (Fig. 1e). Unexpectedly, etoposide had no overt effect on expression of LDHA and MCT1. As suspected, the expression of all of these genes was not altered by etoposide in H446 cells, suggesting the potential involvement of metabolism reprogramming in etoposide resistance onset in NSCLC cells.

We therefore sought to determine whether the metabolic player could mediate etoposide resistance in NSCLC cells. There is accumulating evidence showing that glycolysis-derived lactate plays a crucial role in metastasis and poor prognosis. Etoposide are known to induce replication-dependent DNA double-strand breaks (DSBs) to kill proliferating cancer cells. To get a first insight into a potential involvement of lactate in metabolic regulation of etoposide resistance, we evaluated the etoposide-induced DSBs in lactate-treated cells by examining the levels of H2AX phosphorylation (γH2AX), a surrogate marker for DNA damage, which allowed us to monitor the kinetics of DSB as a direct consequence of metabolic reprogramming. Since it has been reported that the biological effects of lactate may primarily depend on the lactic acidosis in tumor microenvironment in certain conditions [20–22], cells were pretreated with different concentrations of lactic acid (LA) or sodium lactate (NaL) for 3 h before 25 μM etoposide was added. As expected, in both A549 and H1299 lung cancer cells, γH2AX levels was elevated after exposure to etoposide for 36 h (Fig. 1f). Crucially, lactic acid, but no sodium salt, suppressed drug-induced γH2AX levels in a dose-dependent manner, indicating lactic acid is functionally relevant to mediate etoposide-induced resistance. To directly gauge damaged DNA, we next performed a comet assay to detect DSB in the presence and absence of lactic acid. Consistently, the percentage of comet tails was significantly increased in etoposide-treated cells (Fig. 1g), the induction of comet tails by etoposide, however, was dramatically compromised by pretreatment of lactic acid.

Our results suggested that lactate is required for DSB clearance, but did not inform us whether the ability of lactate to protect cells from DNA damage is due to prevent the spontaneous generation of DSB or to repair DSB more efficiently. To distinguish these two possibilities, we modulate the experimental setting by exposing cells to etoposide first for 3 h before the addition of lactic acid, if, as previously implicated, lactate promote DSB repair, then drug-induced DNA lesions could still be recovered by exogenous lactic acid irrespective of schedule of time addition. Interestingly, the later addition of lactic acid could not rescue the DSB responding to etoposide treatment, as indicated by sustained γH2AX levels (Fig. 1h), ruling out its direct involvement in the DSB repair machinery.

We then wished to assess the contribution of lactate on chemoresistance of NSCLC cells. For this, we examined the levels of the cleaved Poly (ADP-ribose) polymerase (cleaved-PARP), an important marker of caspase-mediated apoptosis, in the same of line of experimentation (Fig. 1f). we have found that, in A549 cells, lactate significantly suppressed etoposide-induced apoptosis, in contrast, H1299 cells showed minimal induction of apoptotic program in response to etoposide, and lactate barely affects it, as indicated by the similarly low levels of cleaved-PARP. This phenomenon was recapitulated in both Annexin apoptotic assay (Fig. 1i) and cell viability assay (Fig. 1j). Although necrosis was observed after 36 h incubation with etoposide, the levels of necrosis was marginal compared with apoptosis (Fig. 1i), indicating apoptosis is relevant mechanism of etoposide-induced cell death.

Both commonly mutated tumor suppressor genes, ATM and p53, are considered as the master regulators of the DNA damage response and programmed cell death. It has been well presented that ATM is required for the induction of p53-dependent apoptosis, and suppression of ATM activity strongly reduced the number of apoptotic cells [23]. As such, we measured drug-induced ATM activity in the presence of lactate. Interestingly, we found that lactic acid repressed etoposide-induced ATM activity, as judged by the levels of p-ATM, in both p53-proficient A549 and p53-deficient H1299 cells in a dose-dependent fashion even the H1299 cells had less proportion of apoptotic cells (Fig. 1k). Crucially, reconstitution of p53 in H1299 cells rescued apoptotic response to etoposide, as indicated by appearance of increased cleaved-PARP levels (Fig. 1l). Importantly, lactic acid inhibited etoposide-induced apoptosis in a manner that was ATM dependent. These results indicate that both ATM and p53 are specially required for etoposide induced programmed cell death in NSCLC cells and lactate confers a potent chemoresistance by increasing DSB clearance.

### Induction of snail mediates lactate-induced chemoresistance

**Fig. 2:**
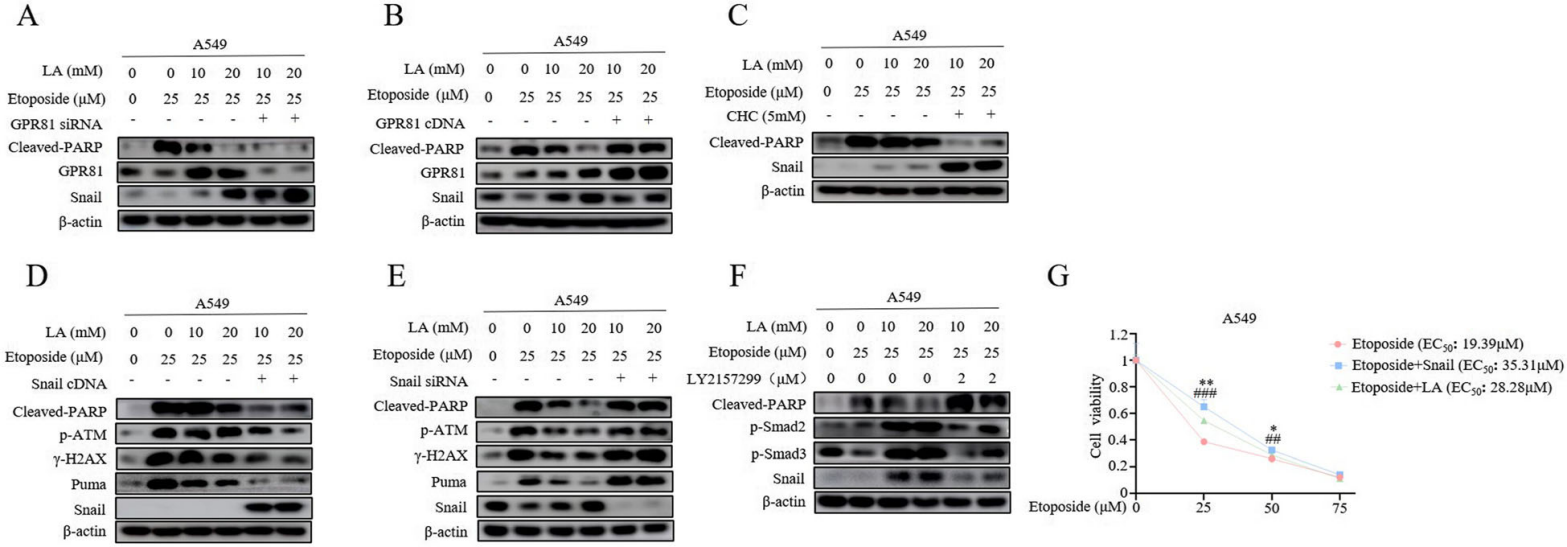
Induction of Snail mediates lactate-induced chemoresistance. **a** and **b** 24 h after transfection with either GPR81 siRNA (**a**) or GPR81 cDNA (**b**), cells were stimulated first with LA (10 mM, 20 mM) for 3 h and then with 25 μM etoposide for additional 36 h. The blots have been probed with antibodies against Cleaved-PARP, GPR81, Snail. **c** The cells were pre-treated with MCT1 inhibitor, a-cyano-4-hydroxycin-namate (CHC) or DMSO as control followed by further combined treatment of LA and etoposide. In contrast to Snail levels, western blot demonstrates a significant decrease in levels of Cleaved-PARP in CHC-treated group. **d** and **e** Western blot analysis of Cleaved-PARP, p-ATM, ATM, Snail, Puma in A549 and H1299 cells at day 3 after infection with Snail cDNA (**d**) or Snail siRNA (**e**) and then further treated with LA and etoposide described in the legend to Fig. 1e. **f** The cells were pre-treated with TGF-β inhibitor, LY2157299, and then with the same treatment as Fig. 2c. **g** The **A549** cells were first transfected with Snail cDNA plasmid. At 48 h post-transfection, cells were treated with etoposide in the presence and absence of LA followed by MTT assay. Corresponding EC_50_values for etoposide, Etoposide+Snail and Etoposide+LA are 19.39 μM, 35.31 μM and 28.28 μM respectively. (*p<0.05, **p<0.01 for difference of overexpressed Snail from etoposide-treated cells, ^#^*p*<0.05 for difference of LA-treated from etoposide-treated cells by ANOVA with Dunnett’s correction for multiple comparisons, ns means no statistical difference)

We next sought to address the mechanism by which lactate promotes chemoresistance. We initially tested the involvement of GPR81, a G protein-coupled receptor for lactate and is critical for cancer survival and progression, to etoposide-induced apoptosis. In stark contrast with our prediction, GPR81 depletion by siRNA did not sensitize A549 cells to etoposide rather strongly enhanced lactic acid-induced chemoresistance, as judged by the levels of cleaved-PARP (Fig. 2a). Furthermore, overexpression of GPR81 restored sensitivity to etoposide treatment (Fig. 2b), suggesting that lactic acid does not transduce its anti-apoptotic signal through GPR81. In addition to stimulation of GPR81, extracellular lactate also contributes acidic microenvironment, both of which all induce malignant phenotypes. Thus, we used the inhibitor of monocarboxylate transport 1 (MCT1), a major transport for the uptake of lactate into cells, a-cyano-4-hytroxyciniamate (CHC) to increase extracellular acidification. Surprisingly, lactic acid induced potent chemoresistance response in the presence of 5 mM CHC (Fig. 2c). These results collectively support the notion that the acidic environment plays an important role in lactate-induced chemonresistance in NSCLC cells.

Prompted by this observation, we tested whether the Snail protein is required for chemoresistance induced by lactate. Snail protein has prominent and widespread roles in activation of EMT, resistance to apoptosis and tumor recurrence. Importantly, induction of Snail protein has been previously documented to be associated with extracellular acidosis [11, 24]. As expected, Snail was upregulated by lactic acid (Fig. 2a and b, compare lanes 3, 4 and lanes1, 2). Intriguingly, combination treatment with lactic acid and CHC not only strongly inhibited etoposide-induced apoptosis but also potently induced Snail protein levels (Fig. 2c, compare lanes 3, 4 and lanes 5, 6), suggesting Snail expression levels positively correlated with status of chemoresistance. Remarkably, overexpression of Snail significantly enhanced lactic acid-induced chemoresistance, as indicated by diminished cleaved-PARP levels (Fig. 2d). Notably, this occurred with a parallel robust decrease of p-ATM and γH2AX levels. In contrast, Snail depletion by siRNA antagonized lactic acid-induced inhibition of apoptosis and levels of p-ATM, and restored sensitivity to etoposide treatment (Fig. 2e).

The p53 transcriptional targeted gene Puma has previously been identified as a critical effector of ATM-p53-dependent proapoptotic program in response to chemotherapy. Interestingly, we found that etoposide-induced Puma expression was suppressed by lactate treatment, and was abolished by concomitant expression of Snail protein (Fig. 2d), Strikingly, depletion of Snail rescued the Puma expression inhibited by lactic acid (Fig. 2e, compare lanes 4 and lanes 6). These data reinforce the notion that induction of Snail by lactate is functionally relevant for lactate-induced chemoresistance.

Our previous work has demonstrated that lactic acid could remodel ECM and release the activated TGF-β1 to induced Snail expression [11], therefore, we examined the impact of inhibition of TGF-β1 pathway on the apoptosis induced by lactic acid. As shown in Fig. 2f, the robust induction of Snail protein was diminished by co-incubation with the TGF-βRI-specific inhibitor LY2157299. This was paralleled by decrease in levels of p-SMAD2/3. Strikingly, addition of LY2157299 sufficiently rescued the apoptosis of lactic acid-treated A549 cells. Consistently, overexpression of Snail increased viability of etoposide-treated cells to a similar extent of lactic acid exposure (Fig. 2g). Taken together, our results reveal that lactate induces Snail expression to promote chemoresistance.

### Lactate confers a potent chemoresistance through upregulation of MRP1

**Fig. 3:**
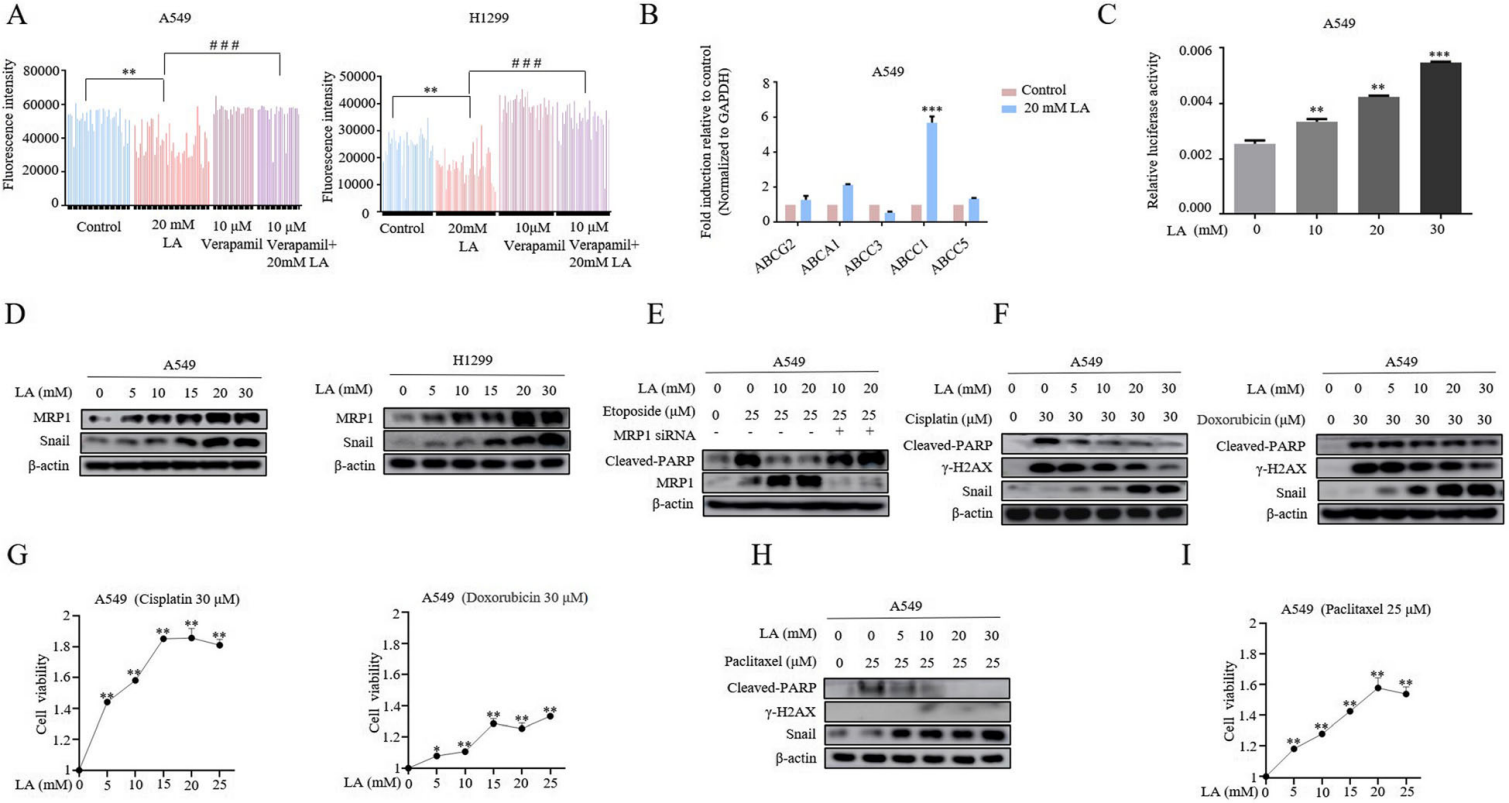
Lactate confers a potent chemoresistance through upregulation of MRP1. **a** Hoechst 33342 efflux assays of cells treated with 20 mM LA in the presence and absence of ABC transporter inhibitor 10 μM Verapamil using BioTek citation 5 (BioTek, Winooski, VT). Representative histogram plots are shown of non-treated cells (control), 20 mM LA, 10 μM Verapamil and combined treatment of lactate and Verapamil. The result clearly showed that lactate significantly promoted the efflux of Hoechst 33342 in A549 and H1299 cells (**P<0.01, ***p<0.001 for difference from untreated control, ^###^*p*<0.001 for difference from LA-treated cells by ANOVA with Dunnett’s correction for multiple comparisons). **b** Quantitative real-time PCR was performed for analysis of mRNA expression levels of several members of ABC transporter family. Values represent the relative increase of MRP1 mRNA normalized to GAPDH. (***p<0.001 for difference from untreated control by ANOVA for multiple comparison). **c** A549 cells were co-transfected with MRP1 promoter reporter construct and control Renilla luciferase reporter plasmid and treated with indicated lactate concentrations after 48 h. Luciferase activity was determined and normalized using the dual luciferase reporter system (**P<0.01, ***p<0.001 for difference from untreated control by ANOVA for multiple comparison). **d** Western blot demonstrates increased MRP1 expression following 3 h of LA (0, 5, 10, 15, 20 and 30 mM) stimulation in both A549 and H1299 cells. **e** 48 h after transfection of A549 either with MRP1 siRNAs or control siRNA, and then cells were stimulated first with LA for 3 h and further etoposide for additional 36 h before western blotting. The results showed that the apoptosis marker Cleaved-PARP was significantly induced after knockdown of MRP1. **f** A549 cells were stimulation with different doses of LA for 3 h and then further exposed to indicated drugs for additional 36 h, western blot results show that LA can significantly reduce Cisplatin, Doxorubicin-induced levels of Cleaved-PARP and γ-H2AX. **g** Cell viability assays show that LA can significantly increase A549 cells survival in the presence of cisplatin (30 mM) and doxorubicin (30 mM). **h** The cells were treated as described in Fig. 3f, western blot results show that LA can significantly reduce Taxol-induced levels of Cleaved-PARP, but not that of γ-H2AX. **i** Cell viability assays show that lactate can significantly increase A549 cells survival in the presence of Taxol (25 mM)

The results described so far suggested that lactate contributed to etoposide-resistant cell survival by increasing damaged DNA clearance and was dependent on Snail expression. It remained unclear how lactate was involved in DSB clearance. The aforementioned finding that lactic acid-induced decline of DSB was not due to increased repair efficiency raised the possibility that lactic acid might increase rates of drug efflux. To test this hypothesis, we first performed Hoechst efflux assay for examining the efflux of fluorescent dye Hoechst 33342 in lactic acid-treated A549 cells. As shown in Fig. 3a, the ability of lactic acid to efflux the Hoechst dye was demonstrated by showing the decreasing fluorescent intensity in lactic acid-treated cells. Expression of ATP binding cassette (ABC) transporters has been established to contribute to the dye efflux and confer resistance to cytotoxic chemotherapy. To get a first insight into a potential involvement of ABC transporters in lactate-induced chemoresistance, we performed quantitative real-time PCRs to measure the transcripts of several transporters in response to lactic acid treatment. In deciding which transporters to study, we tried to focus the transporters to be involved in NSCLC drugs resistance and etoposide is their substrate. These considerations turned our attention to *ABCG2*, *ABBC1, ABCA1, ABCC3, MRP1ABBC1* and *ABCC5* [25]*,* for which we developed specific PCR primers*.* Strikingly, The RT-qPCR assay showed lactic acid treatment only significantly increased MRP1/ABCC1 mRNA levels (Fig. 3b). Consistent with the observed increases in mRNA levels, the MRP1/ABCC1 promoter activity was also induced by lactic acid, as indicated by a dose-dependent increase in luciferase activity in dual luciferase assay (Fig. 3c). In coincident fashion, lactic acid also dose-dependently increased levels of MRP1 protein expression in tandem with Snail protein in both A549 and H1299 cells (Fig. 3d), we thus focused our attention to role of MRP1 in lactic acid-induced drug efflux. Crucially, depletion of MRP1 by siRNA rescued increased cell apoptosis in lactic acid-treated cells as judged by increased cleaved-PARP levels (Fig. 3e). Moreover, in agreement with the effect of MRP1 in mediating cellular efflux of a variety of xenobiotics, lactic acid-treated cells showed dose-dependent γH2AX level decline upon treatment of established MRP/ABCC substrates Doxorubicin and Cisplatin [25, 26], which are known to cause DNA lesions to induce apoptosis (Fig. 3f). In these experiments, changes in γH2AX levels were consistently paralleled by changes in the rate of cell survival, as indicated by decreased levels of cleaved-PARP (Fig. 3f) and increased cell viability (Fig. 3g). Interestingly, lactic acid had no effect on γH2AX levels in A549 cells exposed to paclitaxel (Fig. 3h), which inhibits microtubule disassembly process to kill proliferating cells [27], but was sufficient to rescue drug-suppressed cell survival (Fig. 3i), consistent with the DNA repair-independent nature of this resistance program induced by lactate. Taken together, the data reveal that lactate promotes chemoresistance through upregulation of MRP1.

### Formation of snail/TAZ/AP-1 complex induced by lactate is required for MRP1 expression

**Fig. 4:**
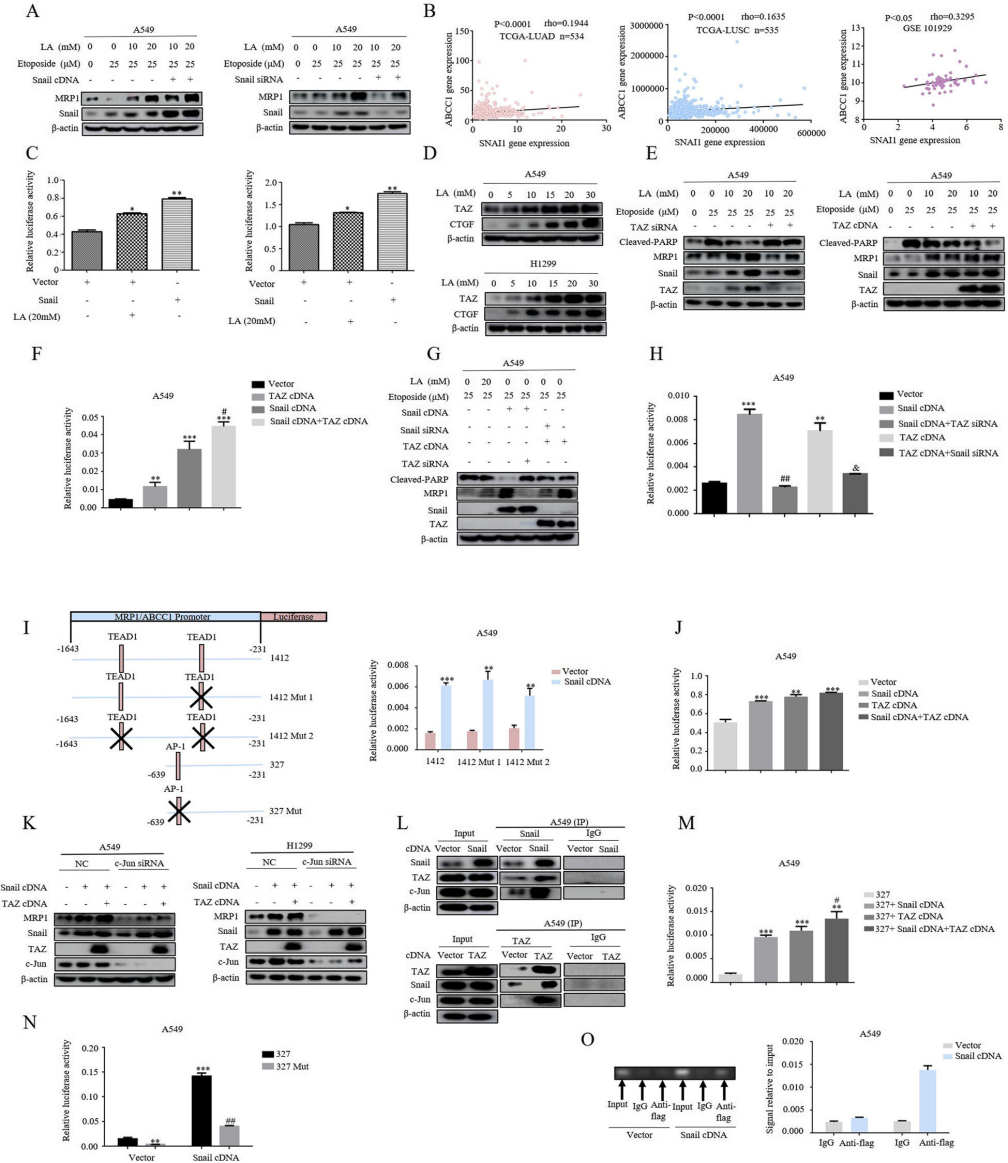
Formation of Snail/TAZ/AP-1 complex induced by lactate is required for MRP1 expression. **a** Transfection of A549 with either Snail cDNA or Snail siRNAs or their respective controls for 48 h, and then were treated first with LA for 3 h and then etoposide for additional 36 h before western blotting. **b** The correlations between SNAI1 and MRP1 expression from three data sets (TCGA-LUAD, TCGA-LUSC, GEO101929). The Spearman’s rank correlation coefficient (rho) and the *P* values were calculated. **c** A549 and H1299 cells were co-transfected with a 8xGTIIC-luc plasmid containing eight synthetic responsive elements of TEAD and Snail cDNA. Then luciferase activity was measured in the presence and absence of LA treatment and normalized using the dual luciferase reporter system. The bars represent the mean ± S.D. of triplicates. (*p<0.05, **P<0.01, for difference from untreated control by ANOVA for multiple comparison, ns means no statistical difference). **d** Western blot demonstrates increased TAZ and CTGF expression following 3 h of lactate (0, 5, 10, 15, 20 and 30 mM) stimulation in A549 and H1299 cells. **e** Transfection of A549 with either TAZ siRNA or TAZ cDNA or their respective controls for 48 h, and then were treated first with LA for 3 h and then etoposide for additional 36 h before western blotting. The results showed that MRP1 was significantly reduced after knockdown of TAZ and increased upon overexpression of TAZ. **f** Cells were co-transfected with MRP1 promoter reporter construct with a Snail cDNA construct, TAZ cDNA construct and combination of Snail cDNA construct and TAZ cDNA construct for 48 h before luciferase activity was determined and normalized. Data are represented as mean ± S.D. (*p < 0.05, **p < 0.01, ***p < 0.001, for difference from cells transfected with control vector, ^#^*p*<0.05 for difference from Snail cDNA-transfected cells by ANOVA with Dunnett’s correction for multiple comparisons). **g** Western blot analysis of MRP1 expression after co-transfection of cells with Snail cDNA or siRNA or TAZ cDNA or siRNA for 48 h upon treatment described in Fig. 4e. **h** Cells were co-transfected with MRP1/ABCC1 promoter reporter construct with a wild-type Snail cDNA or TAZ cDNA or the combined Snail cDNA and TAZ siRNA or the combination of TAZ cDNA and Snail siRNA for 48 h before luciferase activity was determined and normalized. Data are represented as mean ± S.D. (*p < 0.05, ***p < 0.001, for difference from the untreated cells; ^####^*p*<0.001 for difference from cells transfected with Snail cDNA; ^&^*p* < 0.05 for difference from cells transfected with TAZ cDNA by ANOVA with Dunnett’s correction for multiple comparisons)**. i** A schematic representation of the MRP1/ABBC1 promoter region showing potential TEAD1 and AP-1 binding sites (left panel). Cells were co-transfected with one of these designated constructs (constructs 1412, construct 1412 Mut1 with one mutated TEAD1 binding site,1412 Mut2 with mutated two TEAD1 binding sites) and with a wild-type Snail cDNA construct for 48 h, and then luciferase activity was measured and normalized (right panel). The bars represent the mean ± S.D. of triplicates (**p < 0.01, ***p < 0.001, for difference from cells transfected empty vector by ANOVA with Dunnett’s correction for multiple comparisons). **j** The cells were co-transfected with the AP-1-luc plasmids containing the synthetic response element of AP-1(Jun:Jun; Jun:Fos) and the Snail cDNA or TAZ cDNA plasmids. Luciferase activity was then measured and normalized using a dual luciferase reporter system. Bars represent the mean ± S.D. Triplicate (**p < 0.01, ***p < 0.001, for difference from the cells transfected with control vector by ANOVA with Dunnett’s correction for multiple comparisons). **k** A549 and H1299 were co-transfected with c-JUN siRNA and Snail or TAZ cDNA constructs for 48 h. The blots have been probed with indicated antibodies. **l** A549 cells were transfected either with Snail cDNA or TAZ cDNA for 48 h. Cell lysates were subjected to immunoprecipitation with anti-Snail (upper panel) or with anti-TAZ (lower panel), immunoprecipitates were run in Western blots for Snail, TAZ and c-Jun. **m** Cells were co-transfected with MRP1 promoter reporter construct 327 with the Snail cDNA or TAZ cDNA plasmids for 48 h before luciferase activity was determined and normalized. Data are represented as mean ± S.D. (***p < 0.001, for difference from the cells transfected with control vector; ^#^*p* < 0.05for difference from cells transfected with Snail cDNA by ANOVA with Dunnett’s correction for multiple comparisons). **n** Cells were co-transfected with one of two designated constructs (construct 327, 327 Mut with the mutated AP-1 binding site) and Snail cDNA plasmids for 48 h, and then luciferase activity was measured and normalized. The bars represent the mean ± S.D. of triplicates (**p < 0.01, ***p < 0.001, for difference from the cells transfected with construct 327 cells, ^##^*p*<0.01 for difference from 327Mut-transfected cells by ANOVA with Dunnett’s correction for multiple comparisons). **o** A549 cells were transfected with Flag-tagged Snail cDNA for 72 h. ChIP assays were performed using anti-FLAG antibody. The Standard PCR products were run and scanned (left panel). The histogram was presented as quantification of the PCR results (right panel)

We next explored the molecular mechanism by which lactate regulates MRP1 Expression. The finding that lactate induced Snail-dependent chemoresistance suggested the possibility of Snail-controlled MRP1 expression. Consistent with our hypothesis, silencing of Snail significantly decreased lactic acid-induced MRP1 protein levels in etoposide-treated cells. Conversely, forced expression of Snail led to a remarkable enhancement in MRP1 expression induced by lactic acid (Fig. 4a), supporting the notion that Snail activates MRP1 expression. This notion was reinforced by analysis of gene expression data obtained from the Cancer Genome Atlas (TCGA) and Gene Expression Omnibus (GEO) database, revealing a significant positive correlation between Snail and MRP1 (Fig. 4b). However, the attempt to dissect the molecular mechanism by which Snail directly activates MRP1/ABCC1 gene expression was precluded by the bioinformatic analysis that there are no putative Snail binding sites in the MRP1/ABCC1 promoter region. However, the recent study that Snail could form complex with TAZ [28], a transcriptional co-activator, to activate transcriptional events prompted us to examine whether TAZ was required for lactate-induced MRP1 expression. For this, luciferase activity assays were performed in cells transfected with 8xGTIIC-luc plasmids, the well-documented readout of TAZ transcriptional activity, and we demonstrated that both lactic acid treatment and Snail overexpression significantly increased luciferase activity of 8xGTIIC-luc constructs (Fig. 4c). Furthermore, western blot indicated that TAZ expression levels was dramatically induced by either lactic acid exposure or Snail overexpression, in tandem with CTGF protein levels, the most established transcriptional target of TAZ (Fig. 4d). To further confirm whether TAZ regulates MRP1 expression, we next examined the effect of manipulation of TAZ levels on the expression of MRP1. Lactic acid dose-dependently increased MRP1 expression in the presence of etoposide in A549 cells, crucially, this induction was dramatically enhanced in cells overexpressing TAZ protein (Fig. 4e), in contrast, TAZ knockdown by siRNA significantly attenuated lactic acid-induced MRP1 levels. Next, we investigated its relationship with Snail on the MRP1 expression. As expected, each of the cDNAs markedly increased activity of MRP1/ABCC1 promoter in dual luciferase reporter assays, importantly, the combination of Snail and TAZ overexpression induced a synergistic effect (Fig. 4f). The induction of MRP1 expression by each of the cDNAs was also observed in Western blot analysis (Fig. 4g, compare lanes 1, 3, 6), strikingly, depletion of TAZ significantly attenuated Snail-induced MRP1 levels (Fig. 4g, compare lanes 3, 4), likewise, the induction of MRP1 expression by TAZ was reversed by knockdown of Snail (Fig. 4 g, compare lanes 5, 6). Similar results were obtained by the MRP1 promoter luciferase reporter assays (Fig. 4h). These epistatic relationships reinforce the notion that both Snail and TAZ were essential mediators of lactate-induced MRP1 expression.

TAZ functions as co-activator for several transcriptional factors, mainly with the members of TEAD family. The ability of Snail to promote TAZ transcriptional activity led us to postulate that Snail might function by bridging TAZ to the TEAD transcriptional factors. In this scenario, mutation of TEAD binding sites in MRP1/ABCC1 promoter should be insensitive to Snail-induced MRP1/ABCC1 promoter activity. In stark contrast with our prediction, mutation of either one or two putative TEAD1 binding sites has no any effect on the Snail-induced activity of MRP1/ABCC1 promoter (Fig. 4i), suggesting that a mechanism entailing directing regulation of MRP1 expression by TEAD transcriptional factors was unlikely.

As TAZ can also associate with AP-1, and AP-1/TAZ complexes activate targeted genes directly involved in oncogenic growth [29]. The bioinformatic analysis of MRP1/ABCC1 promoter region identified two potential targeting sites for AP-1. Importantly, the transcriptional activity of an AP-1 responsive luciferase reporter construct was significantly elevated by either overexpression of Snail or TAZ as well as the two combination (Fig. 4j). We then sought to determine whether AP-1 is required for Snail/TAZ-induced MRP1 expression. AP-1 are composed of dimers of Jun and Fos families of leucine-zipper proteins, since Jun is a common component of Jun/Jun and Jun/Fos dimers, we thus considered Jun as a surrogate for bound AP-1 dimers. We found that knockdown of c-Jun abrogated the inductive effect of Snail and TAZ on MRP1 expression in both A549 cells and H1299 cells (Fig. 4k). As such, we considered a model wherein Snail/TAZ form complexes with c-Jun to control MRP1 expression. Indeed, following overexpression of epitope-tagged TAZ or epitope-tagged Snail, interacting complexes between Snail, TAZ and c-Jun were detected by-co-immunoprecipitation in A549 cells (Fig. 4l). To further explore the role of this complexes in modulation of MRP1/ABCC1 promoter activity, deletion constructs were tested in the promoter luciferase reporter assays. Constructs contains one predicted AP-1 binding site (327) still showed significant induction of Snail-induced MRP1/ABCC1 promoter activity (Fig. 4m), suggesting an essential role for this AP-1 putative binding site. Crucially, the construct 327 with point mutations in this binding site abolished the responsiveness of the promoter activity to Snail overexpression (Fig. 4n). Moreover, Chromatin immunoprecipitation (ChIP) analysis confirmed the presence of Snail in the MRP1/ABCC1 promoter regions covering the last AP-1 binding site (Fig. 4o). These results together indicated that the formation of Snail/TAZ/AP-1 complex in response to lactate is required for MRP1 expression.

### HIF-1α is a key regulator of drug-induced metabolic reprogramming

**Fig. 5:**
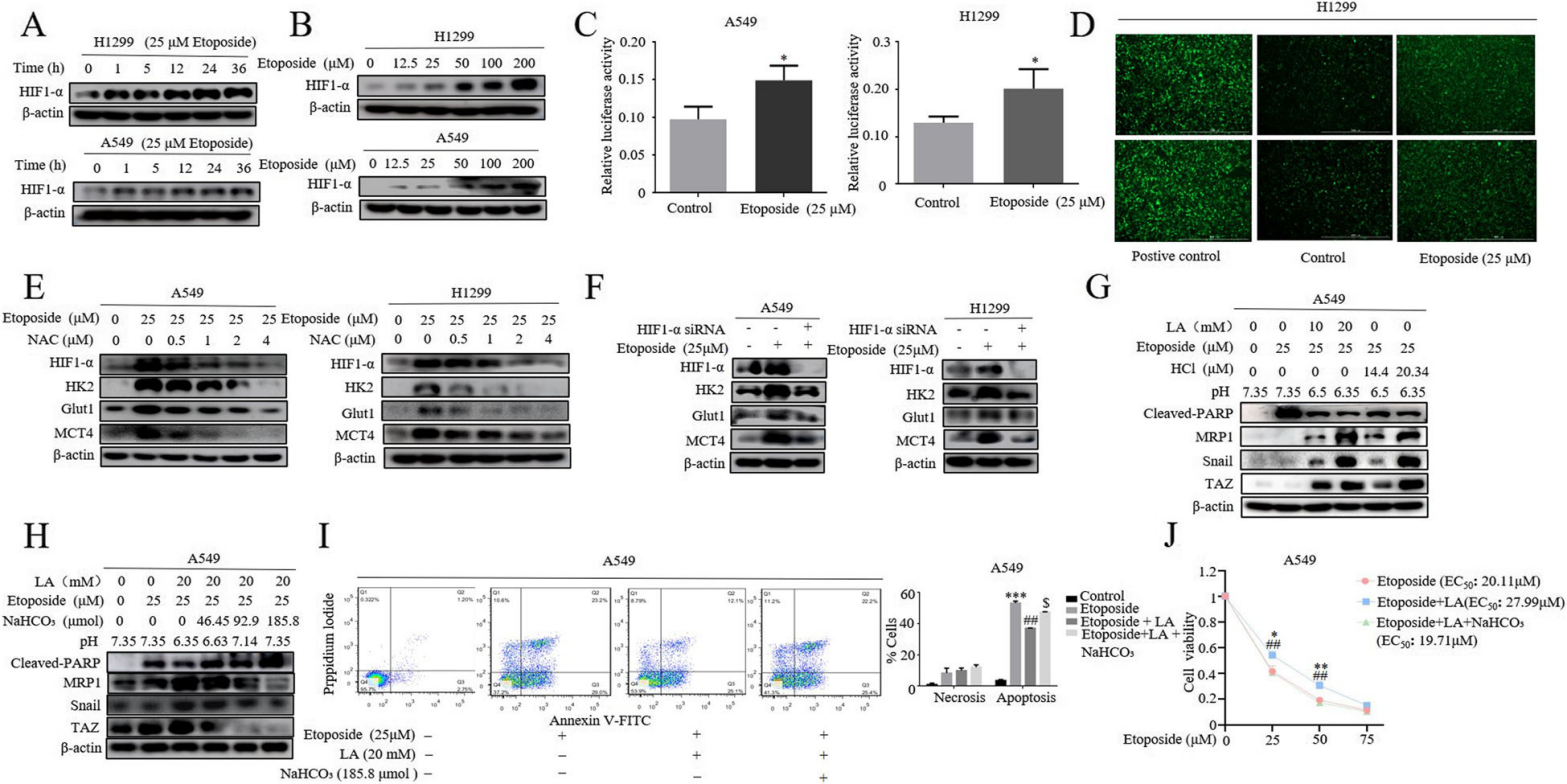
HIF-1α is a key regulator of drug-induced metabolic reprogramming. **a** and **b** Western blot demonstrated the time-course (**a**) and dose-dependent (**b**) induction of HIF1-α by etoposide. **c** A549 and H1299 cells were transfected with a HRE-luc plasmid containing five synthetic responsive elements of HIF1-α for 24 h and then treated with etoposide for 5 h. Then luciferase activity was immediately measured and normalized using the dual luciferase reporter system. The bars represent the mean ± S.D. of triplicates (*p < 0.05 for difference from untreated cells by ANOVA for multiple comparison). **d** A549 Cells were treated with etoposide for 5 h before determination of ROS. **e** A549 and H1299 cells were pretreated with increasing concentrations of N-acetylcysteine (NAC) for 30 min before 25 μM etoposide was added. Western blot was conducted 5 h later to determine levels of HIF-1α, HK2, MCT4, Glut1. **f** A549 cells were transfected with HIF-1α siRNA for 24 h and further treated with 25 μM etoposide for 36 h. Western blot was performed to examine the expression of HK2, MCT4, Glut1. **g** The cells were first stimulated either with different concentrations of LA or different degree of acidification by adding HCl into medium for 3 h followed by further treatment with 25 μM etoposide for additional 36 h and then lysed for western blot analysis of MRP1, cleaved-PARP and Snail expression. **h** Gradual titration of NaHCO_3_ to LA-rich medium dramatically sensitize A549 cells to etoposide treatment as indicated by Cleaved-PARP levels. **i** Cell apoptosis assay. Addition of NaHCO_3_ rescued the etoposide-induced apoptosis inhibited by lactate treatment by Annexin-PI dual staining. The quantification was presented in right panel (**p < 0.01, ***p < 0.001, for difference from the untreated cells, ^##^*p*<0.01 for difference from etoposide-transfected cells, ^$$^*p* < 0.01 for difference from the combined etoposide and LA treated cells by ANOVA with Dunnett’s correction for multiple comparisons) **j** Cell viability assay showed titration of lactic acid with NaNCO_3_ rescued the etoposide-induced inhibition of cell viability. Corresponding EC_50_ values for etoposide, Etoposide+LA and Etoposide+LA + NaNCO_3_ are 20.11 μM, 27.99 μM and 19.71 μM respectively

Since the acquired resistance to etoposide was strongly linked to increased lactate production, we finally wished to understand how etoposide regulated the glycolysis in NSCLC cells. HIF-1α is a master transcriptional factor that controls tumor metabolic reprogramming, given that HIF-1α target genes HK2, Glut1 and MCT4 [30], major enzymes of glycolytic pathway, were elevated in etoposide-treated NSCLC cells (Fig. 1c), we explored whether etoposide also impacted HIF-1α expression. Remarkably, etoposide increased HIF-1α levels in a dose- and time-dependent manner (Fig. 5a, b). Importantly, HIF-1α activity was also induced by etoposide as indicated by HIF-1α response element (HRE) luciferase reporter assay (Fig. 5c). We previously showed that reactive oxygen species (ROS) could mimic the effect of hypoxia to induced HIF-1α expression and activity [31]. Consistent with the findings that etoposide not only induced DSB but also stimulated generation of ROS [32], elevated ROS levels was evident in etoposide-treated H1299 cells (Fig. 5d). We then asked whether the ROS was involved in etoposide-induced upregulation of HIF-1α. The cells were pretreated with ROS scavenger N-acetyl-L-cysteine (NAC), and then exposed to etoposide for 3 h. As shown in Fig. 5e, HIF-1α induction by etoposide was markedly attenuated by NAC in dose-dependent manner in both H1299 and A549 cells. Crucially, siRNA-mediated knockdown of HIF-1α diminished etoposide-induced expression of HK2, Glut1 and MCT4 (Fig. 5f), indicating HIF-1α is a key regulator of etoposide-induced metabolic rewiring.

Our finding that lactate derived from tumor metabolic reprogramming plays a crucial role in chemoresistance. To demonstrate the biological relevance of our finding, we asked whether MRP1 expression is also pH-dependent and titration of the culture medium with NaHCO3 could sensitize cancer cells to etoposide treatment. As expected, addition of lactic acid into culture medium shifting pH from 7.35 to acidic pH caused a robust increase in MRP expression with a parallel rise of Snail protein (Fig. 5g). Notably, adjusting of pH by adding HCl to the corresponding acidification significantly increased levels of MRP expression to a similar extent (Fig. 5g). Consistently, lactic acid treatment resulted in significant inhibition of apoptosis, as indicated decreases in Cleaved-PARP levels in the etoposide-treated cells, in tandem with up-regulation of MRP1, Snail and TAZ expression, (Fig. 5h, compare lanes 1, 2, 3). Remarkably, gradual addition of NaHCO_3_ into culture medium shifted from acidic pH 6.63 to basic pH 7.35 dramatically rescued the phenotypes resulting from lactic acid stimulation (Fig. 5h, compare lanes 4, 5, 6). The similar results were obtained with apoptosis assay (Fig. 5i) and cell viability assay (Fig. 5j). Our findings may suggest a broader benefit of bicarbonate therapy in the treatment of chemoresistant lung cancer patients.

## Discussion

The poor prognosis of advanced lung cancer could partially be explained by robust resistance to chemotherapy. For this reason, enormous efforts have been made to identify the novel genes and signaling networks that are involved in chemoresistance.

Etoposide is an inhibitor of topoisomerase II enzyme that resulted in lethal DNA breakage and has been used more frequently to treated SCLC, but the molecular mechanisms responsible for this difference in drug sensitivity and/or resistance between SCLC and NSCLC remain poorly understood. Kasahara and colleagues found that the expression level and activity of topoisomerase II was higher in SCLC than NSCLC and was correlated with sensitivity to etoposide [33], however, a subsequent study suggested that there was no clear association between levels and activities of topoisomerase II and etoposide sensitivity in the lung cancer [34]. The use of modern genomic and proteomic techniques has identified a diverse range of cell-autonomous mechanisms performed in invitro study of cell lines made resistant by continuous drug exposure [35]. However, no translational or clinical study in NSCLC for etoposide treatment has been initiated based on these results. It is increasingly appreciated that tumor microenvironment plays a crucial role in tumor progression and response to treatment. Many evidences showed that tumor hypoxia contributes significantly to chemotherapy failure [36]. However, it is entirely unknown the role of lactate, also an important indicator of cancer prognosis in tumor microenvironment, in the drug resistance.

Here we report for the first time that the increased lactate production mediated non-cell-autonomous etoposide resistance in NSCLC cells. Mechanistic studies clearly demonstrated ABC transporter MRP1 as a dominant mediator of resistance. We also uncovered that the chemotherapeutic agents such as etoposide induced an exacerbation of ROS production which activates HIF-1α-mediated metabolism reprogramming towards glycolysis. Subsequent studies identified both TGF-β1/Snail and TAZ/AP-1 pathway as the key components of resistance program induced by lactate. Formation of Snail/TAZ/AP-1 complex at the MRP1 promoter provided a rationale for lactate-induced chemoresistance. Moreover, titration of lactic acid with NaHCO_3_ was sufficient to overcome resistance, giving a proof of concept of the clinical relevance of our findings.

Accumulating evidence indicated that acidity in tumor microenvironment has a role in resistance to chemotherapy. It has been postulated that selection of apoptosis-resistant phenotypes and direct effect of ion concentrations is likely to be responsible for low pH-induced chemoresistance [37]. Here, we showed that the increased lactate production, due to metabolic reprogramming induced by drug treatment, activates MRP1 transcription, thus limiting multiple drugs responses. Although the impact of acidic microenvironment induced by lactate on tumorigenesis is well established, it has also become evident that lactate contributes to the tumor progression in many ways. In recent years, the studies from our lab and other labs has demonstrated that lactate plays a key role in immune evasion of cancer, this effect has been shown to be mediated in part through its ability to activate its receptor GPR81, which is highly expressed in cancer cells [13, 16, 38, 39]. Furthermore, there is evidence of lactate as an alternative energy source to fuel oxidative tumor cells [40]. We have shown that activation of AKT pathway involves metabolism lactate in the mitochondria facilitated by MCT-1-mediated transport of lactate into cells [19]. It seems unlikely that all these mechanisms are simultaneously operative in cancer patients. We reason that the specific relevance of these activities of lactate will most likely depend on cellular context. In some situations, or experimental conditions, the biological effects of lactate may primarily depend on the lactic acidosis in tumor microenvironment. It has been reported that acidosis leads to loss of the T-cell function of human that can be restored by buffering the pH at physiological values [20–22]. We have demonstrated that acidification itself is a direct cause of the lactate-induced Snail expression [11]. Moreover, overexpression of Snail and lowing pH increased MRP expression and cell viability, phenocopying lactate stimulation. Importantly, titration of acidic medium with NaHCO_3_ was sufficient to overcome resistance induced by lactate. Therefore, in the context of lactate-induced chemoresistance, the most likely culprit was acidic extracellular environment. The key point is that the identified mechanism of adaptive resistance is not based on selection of genetic or epigenetic alterations preexisting in the tumor cells. Since many conditions can cause a glycolytic switch, our results suggest that the described mechanisms of resistance could develop in a broad range of drugs treatment, further extending the clinical relevance of our findings.

An important question is why SCLC cannot reprogram their metabolism upon drug treatment? Our data demonstrated that NSCLC cells with chemoresistance engage glycolysis-dependent metabolic strategies. Our finding that the metabolism of those resistant cells is specially programmed via ROS-induced HIF-1α underscores the idea that emergence of elevated ROS levels in NSCLC but not in SCLC is a key factor in metabolic adaption in response to drug-induced oxidative stress. In this light, we speculate that the ROS level in SCLC maybe already very high, and cannot be further increased. In accordance with this, it has been recently reported that SCLC cells adopted different metabolic strategies for energy and biosynthesis from NSCLC [41, 42]. In contrast SCLC, NSCLC rely more on glutamine/glutamate metabolism pathway which generate glutathione (GSH) to cope with intracellular ROS levels [42]. Thus, lower GSH levels in SCLC could maintain higher ROS accumulation. Another potential mechanism to be considered is the likely slow kinetics of metabolic rewiring associated with chemotherapy resistance in SCLC. It is well known that SCLC has a typical trajectory of initial favorable responses followed by rapid development of multidrug resistance phenotype within a year. Interestingly, Tripathi et al., [43] recently Demonstrated that chemoresistant SCLC cells exhibit a distinct metabolic phenotype characterized by increased lactate production and MRP1 expression in SCLC patient derived xenografts (PDXs) models. Thus, these data suggest that acquired assistance program in lung cancer cells is uniquely driven by metabolic reprogramming and lactate production. Future studies will be required to determine precisely why NSCLC, not SCLC, rapidly engage the metabolic reprogramming following drug treatment. Dedicated studies are needed to dissect these possibilities.

TAZ and YAP are central mediators of Hippo pathway, which participates in several context-dependent transcriptional programs that control organ size, cell proliferation and stemness. TAZ/YAP do not carry a DNA-binding domain and thus function only through interaction with its transcriptional partners. Interaction with these partners such as TEAD1–4, RUNX, KLF4 and AP-1 promotes tumor growth, metastasis and immune evasion [13, 29]. In line, aberrant TAZ/YAP expression and activity is frequently observed in variety of human cancers including lung cancer [19, 44]. It is well established that Hippo kinases MST/LATS negatively regulate TAZ/YAP, leading to TAZ phosphorylation and degradation in the cytoplasm. However, how extracellular signals regulate this pathway remains unclear. More recently, we have demonstrated that glycolysis-derived lactate efficiently increased the expression and activity of TAZ, but not that of YAP, through upregulation of DNA methyltransferase 1 (DNMT1) [19]. Upregulation of DNMT1 by lactate caused hypermethylation of LATS2 gene promoter, leading to TAZ activation. In this study, we further expanded the landscape of TAZ activity by showing that lactate-induced TAZ activity is required for MRP1 expression and is critical for drug resistance in NSCLC cells.

Ample evidence suggests the expression of ABC transporters, especially the multidrug resistance protein 1 (MDR1), MRP1 (ABCC1) and ABCG2, confer potent resistance to chemotherapy. Our study also showed that glycolysis-derived lactate specifically induced MRP1 expression and activity. Consistent with our findings, it has been reported that MRP protein rather than MDR1 or ABCG2 appears to play a more significant role in Drug resistance in lung cancer [45, 46]. Intriguingly, greater MRP protein and mRNA expression was observed in NSCLC than SCLC [47, 48]. Altogether, these results have potential implications for future chemotherapy for NSCLC, which may be enhanced by use of MRP1 transporter inhibitors.

The coordination of cell metabolism and signaling pathways is considered highly important in determining the root cause of cancer and in combating emerging drug resistance. However, how these separate inputs interact with each other to induce cellular responses remains partially understood. A critical concept emerging from this study is that drug treatment induces a fast adaption of cellular metabolism leading increased lactate production and then consequently induces coherent biological responses. Our findings reveal a key role of lactate to coordinate several signaling pathways in drug resistance program. In doing so, increased lactate production induced activation of Snail and TAZ to control MRP1 expression. The fact that lactate can induced resistance program to several chemotherapeutic agents might have profound therapeutic implications. Since increased lactate levels is also associated with tumor metastasis and poor prognosis, lactate metabolism and/or pathway may reveal potential vulnerabilities suitable for therapeutic targeting.

## Conclusion

We have shown that that chemotherapeutic drug etoposide induced lactate secretion by shifting cellular metabolism toward glycolysis in NSCLC. The increasing lactic acid production in extracellular environment confers a potent chemoresistance through the upregulation of ATP-binding cassette (ABC) transporter MRP1 expression, which contributes to drug resistance by pumping the drugs out of cells. Furthermore, we demonstrated that lactic acid-induced upregulation of Snail and TAZ form complexes with AP-1 transcriptional factor to activate MRP1 expression. Collectively, the results reveal potential vulnerabilities of lactate metabolism and/or pathway suitable for therapeutic targeting.

## Data Availability

The data used or analyzed in this study are available from the corresponding author upon reasonable request.

## Abbreviations

NSCLC: Non-small cell lung cancer (NSCLC)
SCLC: Small cell lung cancer (SCLC)
MRP1: Multidrug resistance-associated protein 1
ABC: Transporter
ATP: Binding cassette transporter
DSB: Double-strand break
ECAR: Extracellular acidification rate
LDHA: Lactate dehydrogenase A
HK2: Hexokinase 2
MCT4: Monocarboxylate transport 4
cleaved-PARP: Cleaved Ploy (ADP-ribose) polymerase
CHC: A-cyano-4-hytroxyciniamate
TAZ: Transcriptional co-activator with PDZ binding domain protein
ChIP: Chromatin immunoprecipitation
